# Effect of Thermal Oxidation of Carbon Nanotubes during Wet Spinning into Fibers Using Sodium Cholate Surfactant in Aqueous Dispersion

**DOI:** 10.3390/ma17143581

**Published:** 2024-07-19

**Authors:** Yun Ho Jeong, Jaegyun Im, Gyeong Hwan Choi, Chae Bin Kim, Jaegeun Lee

**Affiliations:** 1 School of Chemical Engineering, Pusan National University, 2 Busandaehak-ro 63beon-gil, Geumjeoung-gu, Busan 46241, Republic of Korea; 2 Department of Polymer Science and Engineering, Pusan National University, 2 Busandaehak-ro 63beon-gil, Geumjeong-gu, Busan 46241, Republic of Korea; 3 Department of Organic Material Science and Engineering, Pusan National University, 2 Busandaehak-ro 63beon-gil, Geumjeoung-gu, Busan 46241, Republic of Korea

**Keywords:** carbon nanotube fiber, wet spinning, thermal oxidation, oxygen containing functional group

## Abstract

Surfactant-based wet spinning is a promising route toward the eco-friendly production of carbon nanotube fibers (CNTFs). However, currently, the properties of surfactant-based wet-spun CNTFs lag behind those produced by other methods, indicating the need for further understanding and research. Here, we explored the surface characteristics of carbon nanotubes (CNTs) that are advantageous for the properties of CNTFs produced by wet spinning, using sodium cholate as a surfactant. Our finding indicates that appropriate thermal oxidation of CNTs enhances the fiber properties, while excessive oxidation undermines them. This implies that the bonding mechanism between CNTs and sodium cholate involves hydrophobic interaction and π-π interaction. Therefore, it is crucial to preserve a clean surface of CNTs in wet spinning using sodium cholate. We believe our research will contribute to the advancement of surfactant-based wet spinning of CNTFs.

## 1. Introduction

Carbon nanotube fibers (CNTFs) represent a macroscopic form of carbon nanotubes (CNTs), characterized by their interconnected structure held together by van der Waals interactions. The inherent one-dimensional nature of CNTs extends to CNTFs, making them an ideal candidate for applications where the unique properties of CNTs need to be preserved and exploited. Notably, the exceptional mechanical and electrical properties of CNTFs make them highly desirable for various technological applications such as composite materials [1], energy storage devices [2,3], and sensors [4,5,6,7]. 

Presently, there are various methods for producing CNTFs, including wet spinning [8], direct spinning [9], and forest spinning [10]. Among these, wet spinning stands out for its scalability and ability to produce fibers with high packing density and alignment, leading to superior properties. Additionally, wet spinning allows for the fabrication of high-purity CNTFs by utilizing commercially available CNTs after purification or surface treatment. Wet spinning techniques can be classified based on the dispersion method, which includes strong acids [11] or surfactants [8]. While acid-based wet spinning systems have shown impressive performance, they suffer from limitations such as susceptibility to moisture and environmental hazards. On the other hand, surfactant-based wet spinning offers a safer, more environmentally friendly approach. In addition, this method has the potential for the mass production of cost-effective multi-walled carbon nanotube (MWCNT) fibers [12]. However, fibers produced using this method often exhibit inferior properties compared to those produced by direct spinning or acid-based wet spinning.

To enhance the properties of CNTFs, it is essential to disperse a higher concentration of CNTs and ensure stability in dispersion. Research has been conducted on the formation and properties of CNTFs using surfactant-based wet spinning, highlighting the influence of the type of surfactant and the dispersion method [13]. Most studies have utilized sonication to create CNT dispersions in aqueous solutions. Bile salts, which are anionic surfactants, have been reported to adsorb onto the surface of SWCNTs, providing superior stability and dispersibility compared to other cationic, zwitterionic, and non-ionic surfactants [14,15]. Further investigations have been carried out to understand the effects of CNT concentration and dispersion time on the properties of CNTFs in this system [16,17]. The spinnability and properties of CNTFs, contingent on the coagulant in the wet spinning system [18], have also been examined. 

However, the full potential of wet spinning has not yet been realized. Its primary advantage lies in its ability to engineer fiber properties by modifying the intrinsic properties of CNTs, which is unattainable during direct spinning and forest spinning where the synthesis of CNTs and their conversion into fiber are interdependent. This naturally motivates us to manipulate the properties of CNTs independently and investigate their impact on CNTFs. In fact, previous research suggests that acid-based wet spinning processes, when combined with thermal oxidation, can enhance fiber properties. This is achieved by removing surface amorphous carbon and introducing a small amount of oxygen, all while preserving the crystallinity of CNTs [19]. However, attempts to enhance the properties of the final CNT fiber product by modifying the intrinsic properties of CNTs through surface modification in surfactant-based wet spinning have not been reported.

This study investigates the influence of the thermal oxidation treatment of CNTs on the properties of CNTFs produced through surfactant-based wet spinning. We hypothesize that altered surface characteristics of CNTs brought about by thermal oxidation will affect both dispersion and fiber properties in surfactant-based wet spinning. Specifically, we propose that the elimination of amorphous carbon and the reduction in oxygen content on CNT surfaces through thermal oxidation will improve the properties of surfactant-based wet-spun CNTFs. However, prolonged thermal oxidation may lead to the excessive formation of oxygen-containing functional groups on CNT surfaces, which could potentially have a detrimental impact on dispersion and fiber formation.

## 2. Materials and Methods

### 2.1. Material

Commercial single-walled carbon nanotubes (SWCNTs) (TUBALL, OCSiAl, Differdange, Luxembourg) were used as received without further purification. The SWCNTs had a diameter of 1.6 ± 0.4 nm and a length exceeding 5 μm. They possessed a carbon purity of 99 wt% and a specific surface area ranging from 800 to 1600 m^2^/g. This information was provided by the supplier. Sodium cholate (SC), the surfactant, was purchased from Tokyo Chemical Industry (Tokyo, Japan). The coagulant, N,N-dimethylacetamide (DMAc) (99.8%) was purchased from Samchun Chemicals (Seoul, Republic of Korea).

### 2.2. CNT Dispersion

**Figure 1:**
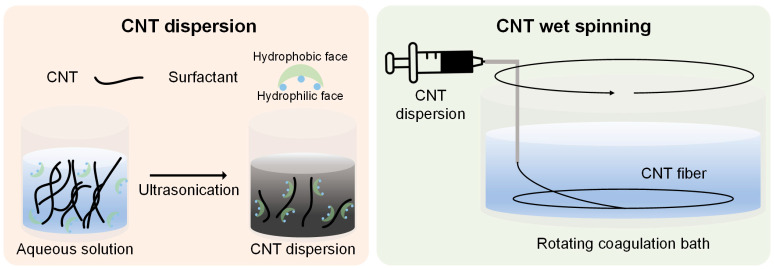
Schematic illustration of CNT dispersion and CNT wet spinning process.

A CNT dispersion of 25 g was prepared by mixing CNTs and the surfactant at a mass ratio of 1:6 in deionized (DI) water (Figure 1). The CNT concentration was 0.4 wt%. The mixture underwent sonication for 30 minutes at 35 °C using a bath sonicator (HWASHIN POWERSONIC 410, 40 kHz, 400 W, Daegu, Republic of Korea) with high power. Subsequently, pre-dispersion was further sonicated for an additional 30 minutes using a probe sonicator equipped with a 6 mm probe (VCX 130, 20 kHz, 130 W, Sonics and Materials, Newtown, CT, USA) operating at a pulse mode of 2 seconds on and 2 seconds off, with the amplitude set at 70%. Following dispersion, CNT aggregates were removed using a vacuum pump equipped with a filter funnel featuring a pore size ranging from 40 to 90 μm. Wet spinning was then conducted after allowing the CNT dispersion to stabilize.

### 2.3. Wet Spinning of CNTFs

Following the transfer of the CNT dispersion into a syringe, the dispersion was injected at a rate of 10 mL/h via a 23 G needle (inner diameter: 337 μm) into a rotating bath operating at 10 rpm. To mitigate gravitational stretching effects, wet spinning was consistently performed with the needle tip positioned 1.6 cm above the floor of the coagulation bath. Following sufficient coagulation time until fiber formation is complete, the fiber was rinsed with deionized (DI) water to eliminate residual surfactant. Subsequently, the fibers were collected in a petri dish and dried overnight at room temperature without applying tension. 

### 2.4. Characterization of CNTs

The Raman spectrum of the CNTs was measured with a laser at an excitation wavelength of 532 nm using a Raman spectrometer (NS240-F, Nanoscope Systems, Daejeon, Republic of Korea). The mean *I*_G_/*I*_D_ of CNTs was calculated from 10 random locations, each with 5% laser power. The *I*_G_/*I*_D_ offers insights into the structural imperfections and defects within CNTs. A higher *I*_G_/*I*_D_ ratio indicates fewer defects. A thermogravimetric analysis (TGA) was performed under nitrogen at a 10 °C/min ramping rate by Discovery TGA 55 (TA Instruments, New Castle, DE, USA). The surface functionalization characteristics of the CNTs were assessed via X-ray photoelectron spectroscopy (XPS) that was performed using an AXIS SUPRA (Kratos Analytical Ltd., Manchester, UK). Transmission electron microscopy (TEM) images were obtained by Titan Cubed 60–300 (FEI company, Hillsboro, OR, USA) with a Cs-Corrector and monochromator at 80 kV. Each sample was dispersed in ethanol by using a bath sonicator (HWASHIN POWERSONIC 410, 40 kHz, 400 W, Daegu, Republic of Korea). Then, the solution was drop-casted on the TEM grid and dried for 4 h at 110 °C in an oven.

### 2.5. Characterization of CNT Dispersions

The state of the CNT dispersion was observed at 40× magnification through an optical microscope (OM) and a polarized optical microscope (POM) (OSH-400PDM, Osun Hitech, Goyang-si, Republic of Korea). A total of 20 μL of CNT dispersion was dropped on the slide glass using a micropipette and the cover glass was gently placed without any pressing. The viscosity of the CNT dispersion was measured using a rheometer (HR20, TA Instruments). The viscosity of the CNT dispersion was measured in a shear rate range of 0.1 to 100 s^−1^ with a gap of 1 mm using a stainless steel parallel plate with a diameter of 40 mm at a temperature of 25 °C.

### 2.6. Characterization of CNTFs

The morphology of the CNTFs was examined utilizing a scanning electron microscope (SEM) (SUPRA 25, Carl Zeiss AG, Oberkochen, Germany). A cross-sectional analysis of the CNTFs was conducted by cutting them using a focused ion beam (FIB) (Helios, Thermofisher Scientific, Waltham, MA, USA). The cross-sectional area of the CNTFs was quantified by employing ImageJ software (1.53e, Java 1.8.0_172 (64-bit)) on the SEM images. Subsequently, accounting for the tilt angle of the fiber at 52°, the precise cross-sectional area was determined by dividing the cosine of 38° by the software-derived area. The linear density of the fibers was computed following a procedure wherein CNT dispersion was injected at a rate of 10 mL/h into a rotating bath operating at 10 rpm for 5 minutes, followed by measurement of the diameter of the circularly stacked fibers. Subsequently, 50 overlapping fibers were weighed post-washing and drying, and the tex (g/1000 m) was ascertained based on the known total length and weight of the fibers.

The mechanical characteristics of the fibers were assessed by employing a universal testing machine (UTM) (DR-100, DrTECH, Bucheon, Republic of Korea), where a tensile test was conducted at a velocity of 12 mm/min utilizing a 1 kgf load cell. All measurement protocols adhered to the ISO 11566:1996 standard [20], with a gauge length of 25 mm and epoxy serving as the adhesive. The resistance (*R*) of the CNTFs was determined by employing the 4-point probe method (Keithley 4200, Tektronix, Beaverton, OR, USA) on a probe station (M5VC, MSTECH, Hwaseong City, Republic of Korea), with 4 cm of spacing between external probes coated with silver paste. Central probes were situated 3 cm apart, with external probes positioned 0.5 cm from the central probe. Electrical conductivity measurements were performed via a current linear sweep operation mode within the range of −0.1 to 0.1 A with a step of 0.005 A. Consequently, the resistivity (*ρ*) and conductivity (*σ*) of the CNTF were computed as follows: $\sigma =\rho^{-1}=\frac{L}{RA}$, where *L* and *A* represent the length and cross-sectional area of the CNTFs, respectively.

## 3. Results and Discussion

### 3.1. Thermal Oxidation of CNTs

**Figure 2:**
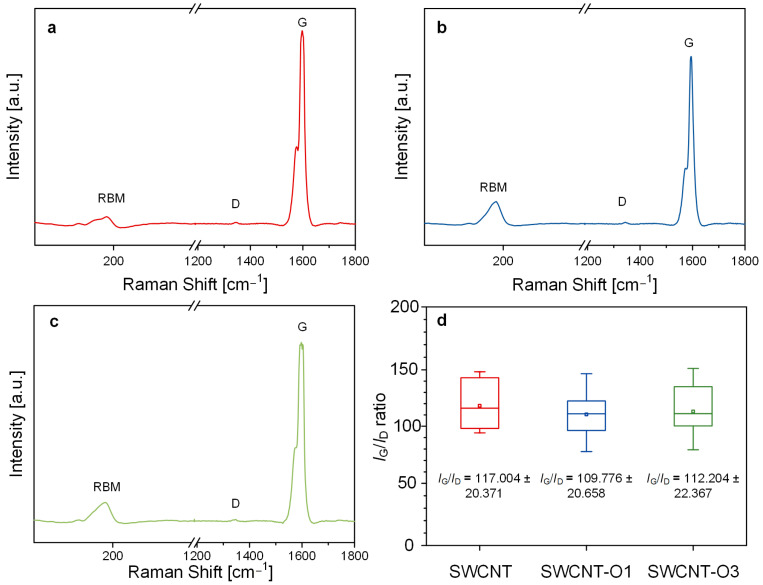
Raman spectra of (**a**) SWCNT, (**b**) SWCNT-O1, and (**c**) SWCNT-O3; (**d**) box plot of *I*_G_/*I*_D_ ratio or each CNT sample. RBM: radial breathing mode; D: D band; G: G band.

In this study, CNTs were utilized without undergoing any additional purification processes. The thermal oxidation of the CNTs was conducted in a tube furnace under an air atmosphere, with the temperature gradually ramping up to 400 °C at a rate of 5 °C/min for durations of either 1 hour or 3 hours (Figure S1). The CNTs that underwent thermal oxidation treatments for 1 hour and 3 hours were designated as SWCNT-O1 and SWCNT-O3, respectively. The sample that underwent no thermal oxidation treatment was designated as SWCNTs. To assess the crystallinity of CNT samples after thermal oxidation treatment, we obtained the Raman spectra for each CNT sample. We performed *t*-tests for each pair of samples and found no significant differences in the *I*_G_/*I*_D_ ratio among the samples. This indicates that the thermal oxidation treatment employed in this study does not significantly alter the crystallinity of CNTs (Figure 2 and Figure S2). The commercially available CNTs had already been subjected to a purification process, resulting in high purity and uniform crystallinity. We also observed the surface state of each CNT through TEM analysis (Figure S3). No significant differences were observed in the TEM images of each CNT after the thermal oxidation process. As explained above, the commercial SWCNTs had high purity because they had already gone through a purification process.

**Figure 3:**
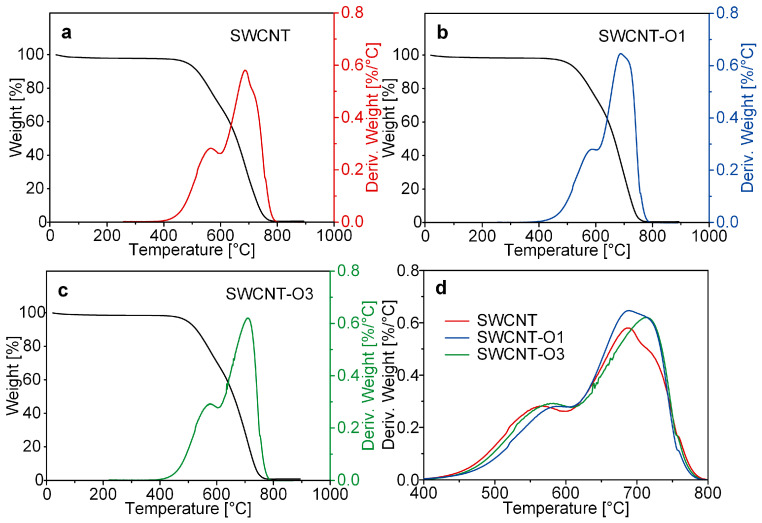
TGA graphs of (**a**) SWCNT, (**b**) SWCNT-O1, and (**c**) SWCNT-O3; (**d**) TGA graph in 400–800 °C.

Although Raman and TEM analyses did not reveal a discernible difference between CNT samples after thermal oxidation, TGA analysis provided more comprehensive insights. The TGA graphs of CNTs allow for the interpretation of the presence of amorphous carbon, oxygen functional groups, and defects based on the areas within the 400–600 °C and 600–800 °C temperature ranges. The amorphous carbon in all CNT samples began to sublimate at 400 °C and underwent complete decomposition at 800 °C (Figure 2a–c). Within the 400–600 °C temperature range, the minimal decomposition observed in SWCNT-O1 implies the least presence of amorphous carbon (Figure 3d). Moreover, the largest graph area in the region above 600 °C indicates a higher proportion of crystalline carbon in SWCNT-O1. The results indicate that the amorphous carbon on the surface of CNTs was effectively removed after one hour of thermal oxidation, which is consistent with a recent report [21]. However, prolonged thermal oxidation created some defects on the wall, likely due to the formation of oxygen-containing functional groups.

To obtain quantitative information about the surface of CNTs, we carried out an XPS analysis. Through the XPS analysis, we aimed to assess the degree of incorporation of oxygen-containing functional groups on the surface of CNTs after thermal oxidation (Table 1 and Figure S4). After an hour of thermal oxidation, the oxygen content decreased from 5.1% to 1.9% and the carbon content increased from 94.5% to 97.3%. This change is attributed to the removal of oxygen functionalities at the amorphous carbon on the surface of SWCNTs during thermal oxidation. As the amorphous carbon was removed, the associated oxygen-containing functional groups were concurrently removed. However, after three hours of oxidation, the oxygen content increased to 7.4%. This increase in oxygen content, concurrent with the progression of thermal oxidation, suggests the introduction of oxygen-containing functional groups onto the surface of CNTs during the thermal oxidation process.

The analysis results indicate that the initial one-hour thermal oxidation process effectively eliminated the amorphous carbon present on the surface of CNTs. Subsequently, the extended two-hour thermal oxidation process led to the introduction of numerous oxygen-containing functional groups on the CNTs’ surface. It is well known that thermal oxidation induces alterations in the surface characteristics of CNTs. A recent study claimed that thermal oxidation is effective in removing amorphous carbon from the surface of CNTs, and that high oxygen content after thermal oxidation is due to iron catalyst oxidation [21]. However, in this paper, we confirmed the absence of metal impurities in pristine SWCNTs by TGA and XPS data. The increase in oxygen content observed in SWCNT-O3s was attributed to the incorporation of surface functional groups rather than to metal oxidation. In the subsequent section, we will elucidate how these altered surface properties impact the dispersion of CNTs, the wet spinning process, and the properties of resultant CNTFs.

### 3.2. Characterization of CNT Dispersion

**Figure 4:**
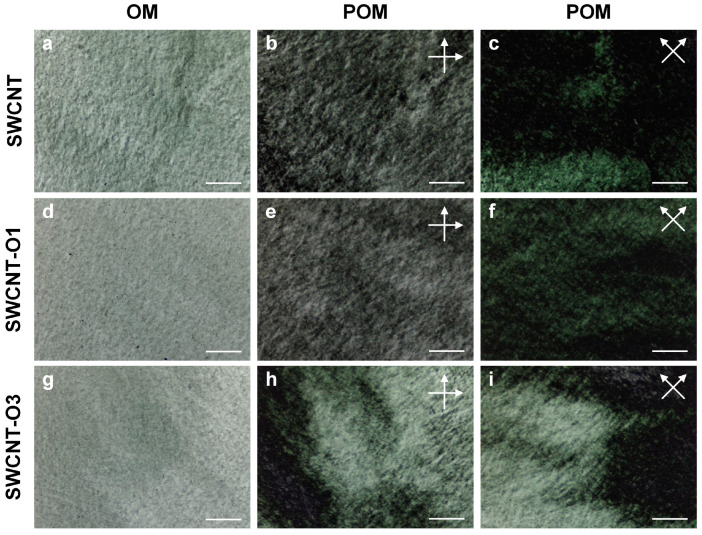
OM and POM images of (**a**–**c**) SWCNT, (**d**–**f**) SWCNT-O1, and (**g**–**i**) SWCNT-O3 dispersion. Scale bars: 200 μm.

For successful wet spinning, CNTs should be well dispersed. We dispersed all the CNT samples in an aqueous solution with a surfactant. The dispersion state of these CNTs was evaluated using POM analysis (Figure 4). Both the thermally oxidized and non-oxidized CNTs formed uniform dispersion states without large aggregates. They also exhibited a liquid crystal phase, which is favorable for wet spinning [22]. 

**Figure 5:**
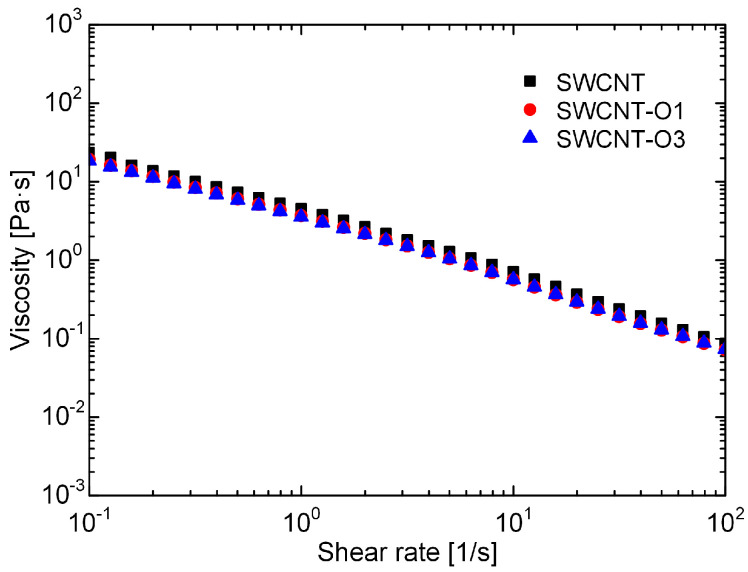
Viscosity of different CNT dispersions at shear rate of 0.1–100 s^−1^.

According to a previous study, CNT dispersions exhibit good spinnability when their viscosity exceeds 10 Pa⋅s at a shear rate of 0.1 s^−1^ [23]. It was observed that all the CNT dispersions, regardless of whether they underwent thermal oxidation treatment, satisfied this criterion (Figure 5). The wet spinning processes further confirmed the formation of stable fibers from these dispersions.

### 3.3. Characterization of CNTFs

**Figure 6:**
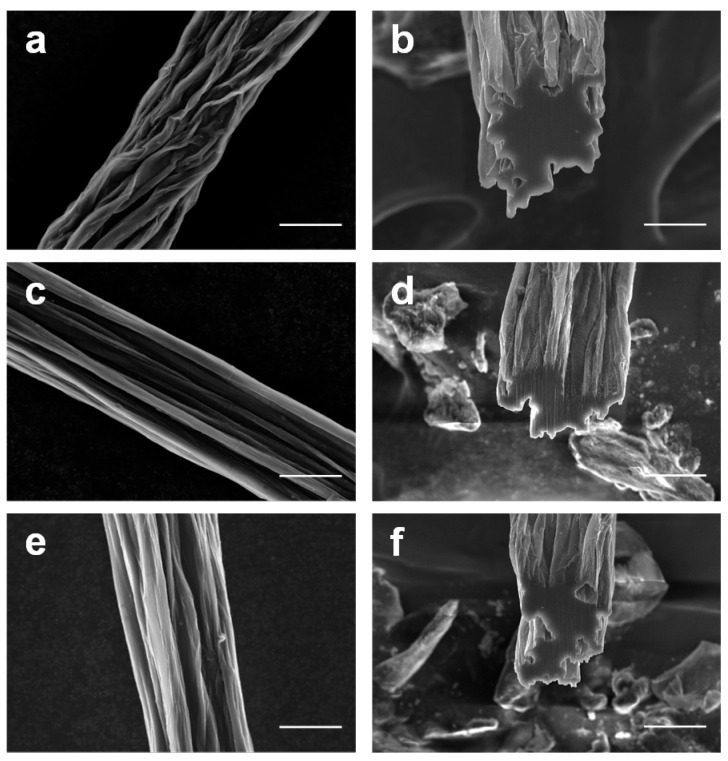
SEM images of CNTFs made of (**a**,**b**) SWCNT, (**c**,**d**) SWCNT-O1, and (**e**,**f**) SWCNT-O3. Scale bars: 20 μm.

From these CNT dispersions, CNTFs were spun using DMAc as the coagulant. The morphology of the surface and cross-section of the as-spun CNTFs were observed by an SEM (Figure 6). In general, the morphology of our CNTFs was similar to those reported in previous studies on surfactant-based CNTFs [24]. It is worth noting that the surfaces of CNTFs spun from thermally oxidized CNTs were significantly smoother than those spun from non-oxidized CNTs. All CNTFs had an approximate diameter of 32 μm and a volumetric density ranging from 0.8 to 1.3 g/cm^3^ (Table S1).

**Figure 7:**
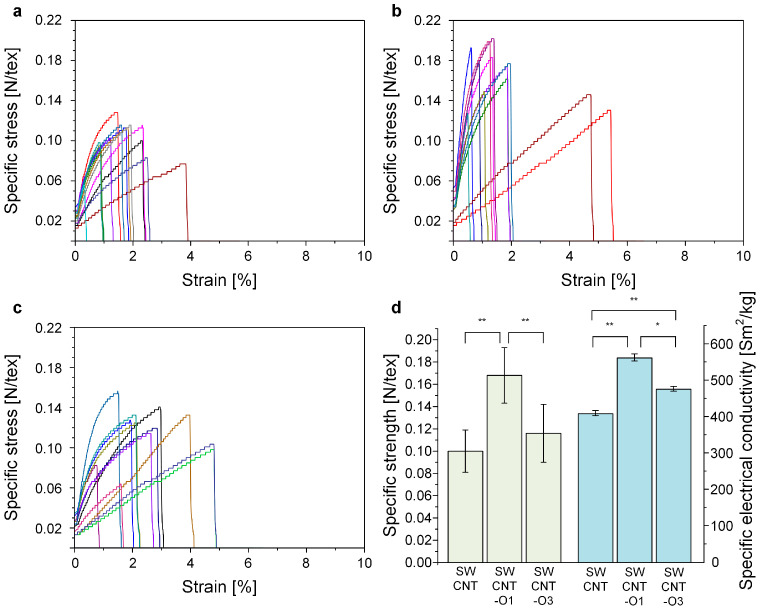
Specific stress–strain curves of (**a**) SWCNT, (**b**) SWCNT-O1, and (**c**) SWCNT-O3; (**d**) specific tensile strength and specific electrical conductivity of CNTFs spun from different CNTs. (*n* = 10 each, * *p* < 0.005, ** *p* < 0.001, *t*-test).

Figure 7 shows the specific stress–strain curves of CNTFs as they varied with thermal oxidation time. The specific strength of CNTFs spun with SWCNT-O1 showed an enhancement compared to the CNTFs spun with pristine SWCNTs. However, the CNTFs spun with SWCNT-O3 showed a lower specific strength compared to those spun with SWCNT-O1. In all CNTFs, the elongation at break scattered from 1 to 5%.

To investigate the effects of thermal oxidation time on CNTFs, the physical and electrical properties of CNTFs were statistically compared using a two-sided *t*-test (Figure 7d). It was observed that both the specific tensile strength and specific electrical conductivity of CNTFs improved after one hour of thermal oxidation but decreased after three hours of treatment when compared to the one-hour oxidation. These results can be interpreted in the context of the interaction between surfactants and CNTs in the dispersion, as well as the interaction among CNTs within the resultant CNTF.

The interaction between SWCNTs and SC has been characterized by hydrophobic and π-π interactions in previous studies [14,25,26]. SC possesses a molecular structure characterized by a rigid carbon backbone with a hydrophobic convex surface and a concave surface containing hydroxyl and carboxylate groups. When dispersing SWCNTs in aqueous solutions, the hydrophobic side of SC tends to adhere to the surface of the CNTs, aiding in the dispersion of the CNTs. Therefore, achieving a highly crystalline surface of CNTs with minimal oxygen-containing functional groups enhances interactions with surfactants. This enhancement, in turn, facilitates the production of uniform dispersions. 

**Figure 8:**
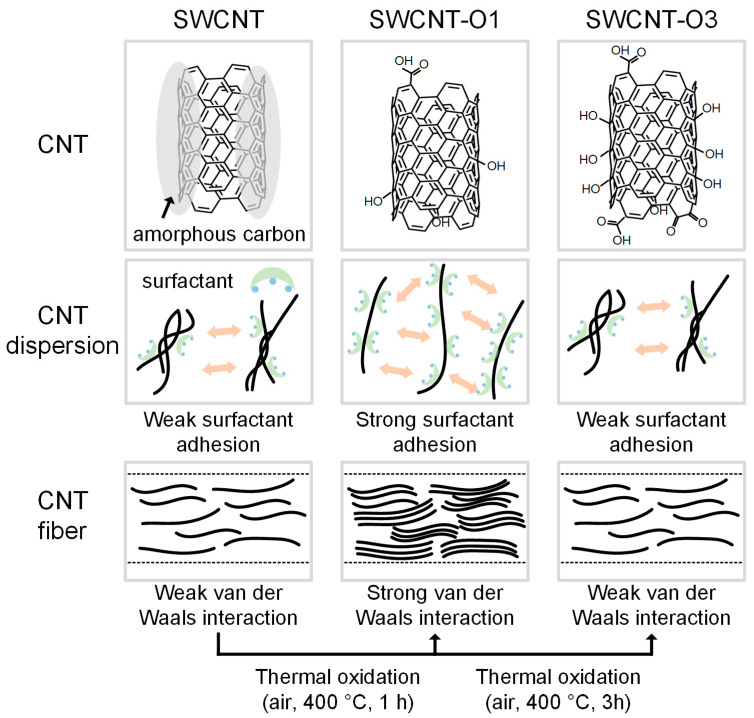
Schematic illustration of CNT, CNT dispersion, and CNT fiber states for each thermally oxidized CNT (SWCNT, SWCNT-O1, and SWCNT-O3).

During the initial phase of thermal oxidation, which lasted for one hour, there was a noticeable removal of amorphous carbon from the surface of the CNTs (Figure 8). Consequently, it can be inferred that the interaction between hydrophobic surfactants and CNTs was strengthened. As a result, a uniform dispersion was achieved, leading to the formation of stronger CNTFs. In contrast, after three hours of thermal oxidation, the increased presence of oxygen-containing functional groups on the surface of the CNTs led to hydrophilic properties. This weakened the interaction between the CNTs and surfactants, thereby impeding the debundling of the CNTs. As a result, the CNTFs exhibited lower alignment and density, resulting in relatively poorer properties. In addition, given that the properties of CNTFs rely on van der Waals interactions between CNTs, CNTs with fewer oxygen-containing functional groups yielded superior properties in CNTFs.

## 4. Conclusions

This study investigated the influence of changes in surface characteristics, brought about by the thermal oxidation of CNTs, on the properties of wet-spun CNTFs using surfactant-based aqueous dispersions, with SC as the surfactant. We observed an enhancement in the mechanical and electrical properties of CNTFs when CNTs, subjected to one hour of thermal oxidation, were utilized. However, these properties deteriorated with longer oxidation duration. We attribute these results to the interaction between the surfactant and the CNTs. The surfactant, SC, adhered to the CNT surface via hydrophobic and π-π interactions. The presence of amorphous carbon and oxygen functional groups on the CNT surface hindered the interaction between CNTs and SC, thereby diminishing the dispersibility of CNTs in the dispersion. Therefore, it is crucial to ensure the surface of CNTs is as clean as possible to achieve the superior mechanical and electrical properties of CNTFs from surfactant-based wet spinning. Our study suggests that the regulation of surface properties is an important strategy for enhancing the properties of CNTFs in the surfactant-based wet spinning process.

## Figures and Tables

**Table 1:** Relative atomic composition (%) of CNTs after thermal oxidation processes, as determined using XPS.

Sample	C1s	Fe2p	N1s	O1s
SWCNT	94.5	0.2	0.2	5.1
SWCNT-O1	97.3	0.4	0.4	1.9
SWCNT-O3	91.9	0.3	0.4	7.4

## Data Availability

The datasets generated during and/or analyzed during the current study are available from the corresponding author upon reasonable request.

## Supplementary Materials

The following supporting information can be downloaded at: https://www.mdpi.com/article/10.3390/ma17143581/s1, Figure S1: Heating condition (a) 1 hour and (b) 3 hours thermal oxidation; Figure S2: Raman spectra of 10 different random sites of (a) SWCNT, (b) SWCNT-O1, and (c) SWCNT-O3; Figure S3: TEM images of (a,b) SWCNT, (c,d) SWCNT-O1, and (e,f) SWCNT-O3; Table S1: The cross-sectional area calculated from SEM images of CNTFs and the volumetric density of CNTFs based on the cross-sectional area. Figure S4: C1s peak spectra of (a) SWCNT, (b) SWCNT-O1, and (c) SWCNT-O3.
