# Supplementary material for: Effect of Thermal Oxidation of Carbon Nanotubes during Wet Spinning into Fibers Using Sodium Cholate Surfactant in Aqueous Dispersion
Source: Materials (Basel). 2024 Jul 19;17(14):3581. doi: 10.3390/ma17143581 (PMC11278946; doi:10.3390/ma17143581)
Supplement: Supplementary file 1 [file materials-17-03581-s001.zip › materials-3091194-supplementary.pdf]

Effect of thermal oxidation of carbon nanotubes on wet spinning into fibers using sodium cholate surfactant in aqueous dispersion

Yun Ho Jeong [1], Jaegyun Im [1], Gyeong Hwan Choi [1], Chae Bin Kim [1,2], and Jaegeun Lee [1,3*]

[1] School of Chemical Engineering, Pusan National University, 2 Busandaehak-ro 63beon-gil, Geumjeoung-gu, Busan 46241, Republic of Korea; dbsgh0919@gmail.com (Yun Ho Jeong)

[2] Department of Polymer Science and Engineering, Pusan National University, 2 Busandaehak-ro 63 beon-gil, Geumjeong-gu, Busan 46241, Republic of Korea; cbkim@pusan.ac.kr (Chae Bin Kim)

[3] Department of Organic Material Science and Engineering, Pusan National University, 2 Busandaehak-ro 63beon-gil, Geumjeoung-gu, Busan 46241, Republic of Korea

* Corresponding author: jglee@pusan.ac.kr (Jaegeun Lee)

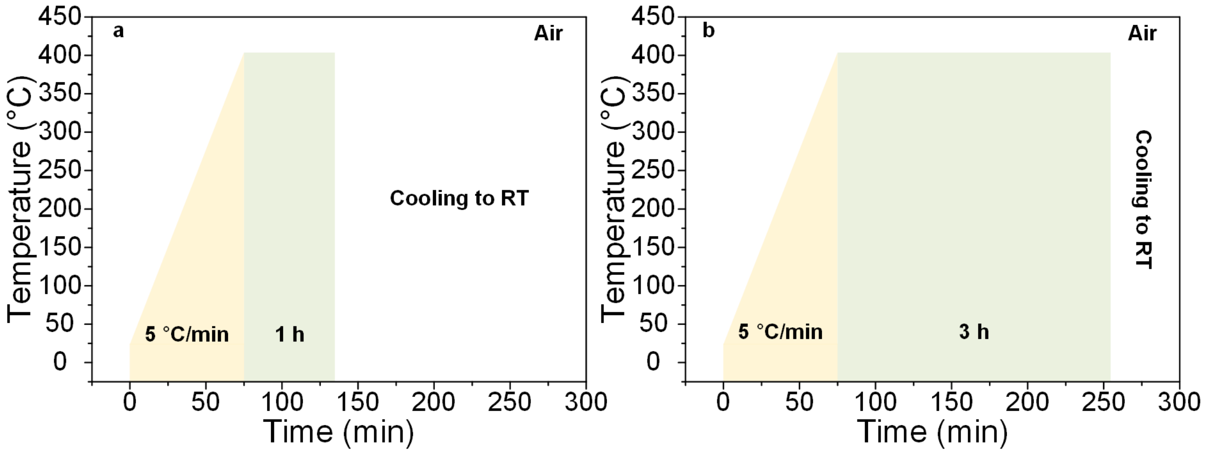

Figure S1. Heating condition of (a) 1 hour and (b) 3 hours thermal oxidation.

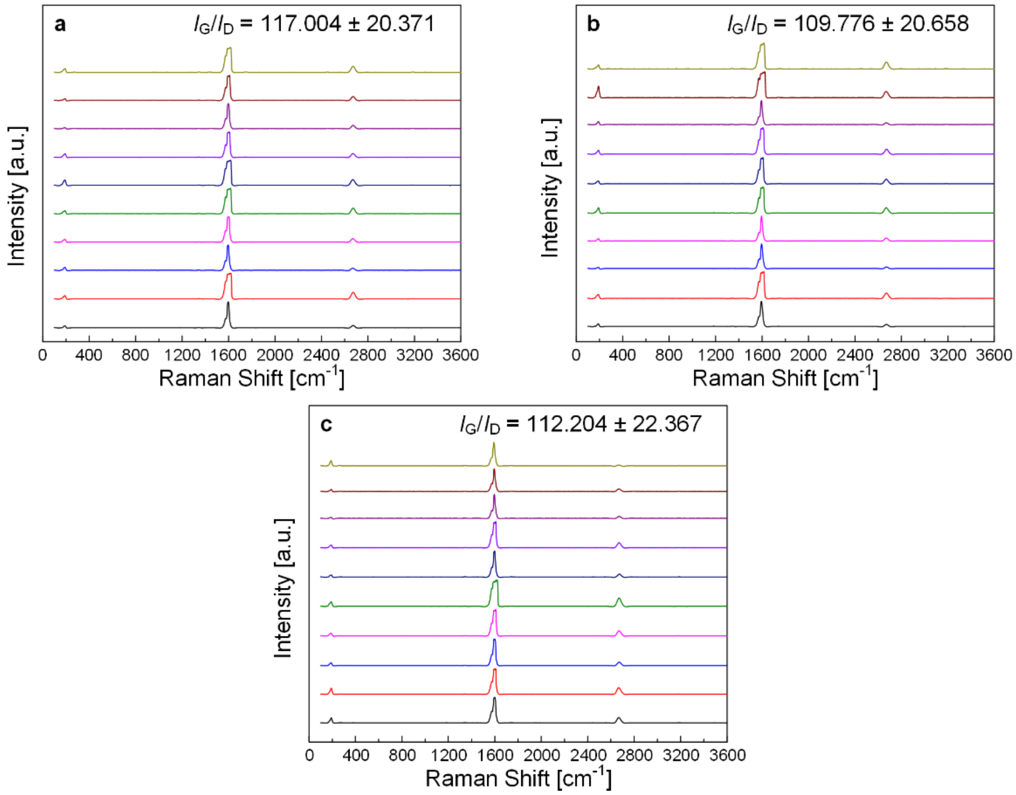

Figure S2. Raman spectra of different 10 random site of a) SWCNT, b) SWCNT-O1, c) SWCNT-O3.

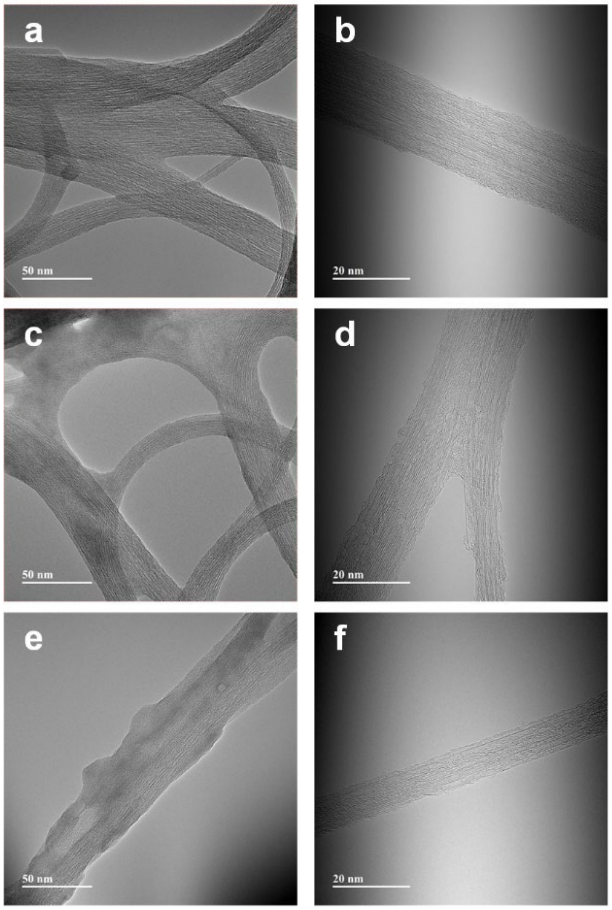

Figure S3. TEM images of a, b) SWCNT, c, d) SWCNT-O1, e, f) SWCNT-O3.

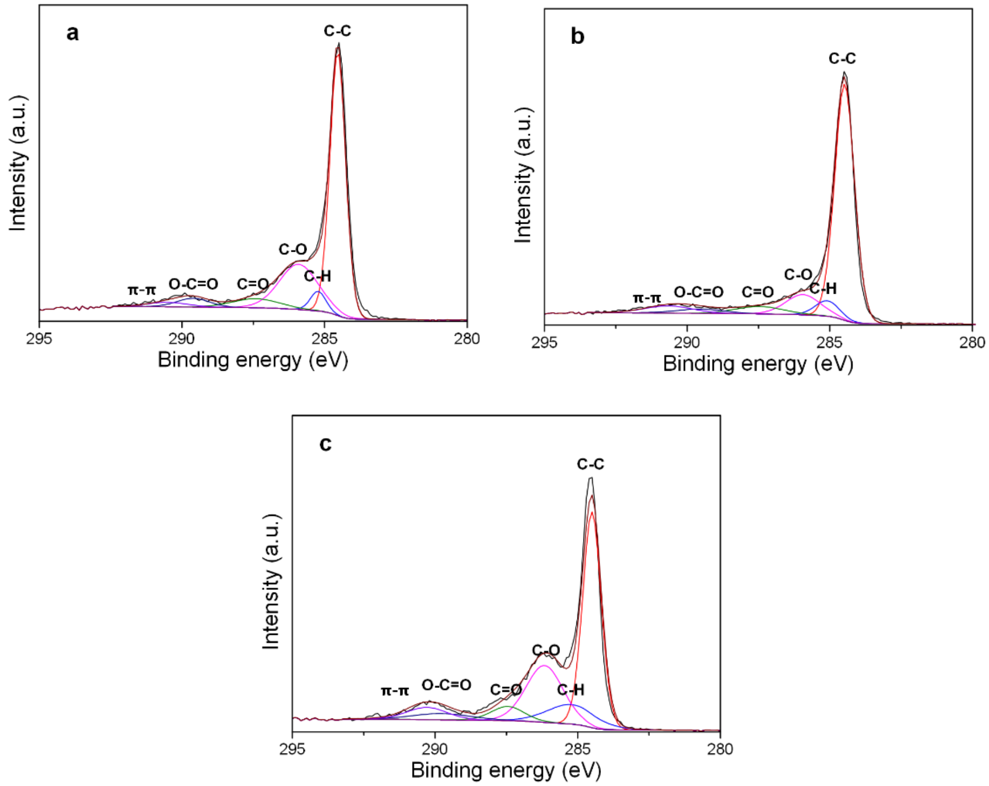

Figure S4. C1s peak spectra of a) SWCNT, b) SWCNT-O1, c) SWCNT-O3.

Table S1**.** The cross-sectional area calculated from SEM images of CNTFs and volumetric density of CNTFs based on cross-sectional area.

CNT fiber	Cross-sectional area [μm^2]	Real cross-sectional area [μm^2]	Tex [g/1000m]	Volumetric density [g/cm^3]
SWCNT	294.872	374.198	0.39	1.14
SWCNT_O1	193.792	245.926	0.322	1.31
SWCNT_O3	361.099	458.241	0.377	0.823
